# Changes in the Bioaccessibility of Antioxidants after Simulated In Vitro Digestion of Bioprocessed Spelt-Enhanced Wheat Bread

**DOI:** 10.3390/antiox12020487

**Published:** 2023-02-15

**Authors:** Marjeta Mencin, Nika Markanovič, Maja Mikulič-Petkovšek, Robert Veberič, Petra Terpinc

**Affiliations:** 1 Department of Food Science and Technology, Biotechnical Faculty, University of Ljubljana, Jamnikarjeva 101, 1000 Ljubljana, Slovenia; 2 Department of Agronomy, Biotechnical Faculty, University of Ljubljana, Jamnikarjeva 101, 1000 Ljubljana, Slovenia

**Keywords:** bioprocessed flour, germination, fermentation, enzymatic treatment, bioaccessibility, in vitro digestion, bread, phenolics, HPLC-MS

## Abstract

The aim of the study was to determine whether the partial replacement of wheat flour with bioprocessed spelt flour contributes to a higher bioaccessibility of the antioxidants in bread. The results showed that the type and amount of bioprocessed spelt flour in a bread recipe has a major impact on the extractable and bound TPC, the content of individual phenolics, their antioxidant activity, and their bioaccessibility as determined by in vitro digestion. Extractable *p*-coumaric and *trans*-ferulic acids in breads decreased after digestion, while extractable *cis*-ferulic and *p*-hydroxybenzoic acids increased. The bioaccessibility of TPC in the control bread (100% wheat flour), and in bread enriched with 5% “germinated + fermented” spelt flour (GFB5), did not differ. However, the digested GFB5 bread contained 5.2-times more extractable, and 1.3-times more bound, *trans*-ferulic acid than the digested control bread. *trans*-Ferulic acid showed the lowest bioaccessibility, up to 2.8%. In GFB2.5 and GFB5 breads, the bioaccessibility of *p*-coumaric, *trans*-ferulic, and *cis*-ferulic acids was higher than in other digested breads. PCA visualized the difference between the undigested and digested breads. The incorporation of germinated and fermented, or germinated and enzymatic, treated spelt flour in a white bread recipe could be an attractive way of providing consumers with nutritionally interesting foods.

## 1. Introduction

Antioxidants in foods play an important role as health protection factors by scavenging free radicals, thus inhibiting the oxidative mechanisms that lead to degenerative diseases. Phenolics are the major antioxidants in cereal seeds, occurring in extractable (free, soluble conjugated) and bound forms [1]. Ferulic acid accounts for up to 90% of the total phenolics in spelt seeds, and 99% of it is present in the bound form [2], which reduces its bioaccessibility [2,3,4,5]. It is important to note that ferulic acid bound to the food matrix cannot be absorbed at the gastrointestinal (GI) level but must be previously released by bacterial enzymes of the microbiota present in the colon [6].

Many bread products, especially those made with whole grains, are a good source of bioactive compounds, including dietary fibre and other phytochemicals [7]. Bioaccessibility is defined as the release of the compound from its natural matrix to be available for intestinal absorption. It has been reported that bioprocessing techniques can increase the bioaccessibility of phenolics from cereal seeds through chemical (mainly oxidation and polymerization) or enzymatic reactions that hydrolyse or release the bioactive compounds from the food matrix [8]. Phenolics in bakery products can be affected by germination [9,10,11], fermentation [2], and enzymatic treatment [12], among other processes. In a previous study, we reported that a combination of germination and fermentation leads to an additive effect, since germination leads to a greater amount of fermentable sources (sugars and nitrogen), while the fermenting microorganisms contribute their own enzymes, increasing the concentration of hydrolytic enzymes, which can lead to the increased bioaccessibility of phenolics [13]. The use of external enzymes in bread-making has also been studied with a view to improving the technological and nutritional properties of the dietary fibre fraction. We recently investigated the combined effects of cellulase, xylanase, feruloyl esterase, α-amylase, and protease on the release of phenolics, and the results showed a significant increase in the extractable content of *p*-coumaric, ferulic, and *p*-hydroxybenzoic acids [12]. Finally, the resulting solubilization of arabinoxylans, and slight degradation of the insoluble fibre contribute to the increased bioaccessibility and antioxidant activity of liberated phenolics [2]. The combination of bioprocessing techniques, therefore, has the potential to improve the nutritional properties of bread by changing the content and/or bioaccessibility of phenolics [8,14].

Cereal products are the most widely consumed staple foods in the world. Among the wide range of products, bread is one of the most consumed. Although the bioaccessibility of bread phenolics has been already researched, most studies have included unprocessed wheat, rye, oat and barley flours [7]. The present research is the first attempt to determine the bioaccessibility of total and individual phenolics from wheat bread enriched with spelt flour, processed with a combination of different bioprocessing techniques, especially because we managed to prove that bioprocessed spelt flour has a more than 2-fold higher content of total phenolics than unprocessed spelt flour [2,12].

The use of bioprocessing techniques would be a good approach to producing bread with an improved nutritional value, and the consumption of bioprocessed bread would have a positive impact on consumer health. The aim of this study was therefore to determine the total phenolic content (TPC), phenolic profile, antioxidant activity, and bioaccessibility of wheat bread enriched with different types and proportions of bioprocessed spelt flour before and after digestion.

## 2. Materials and Methods

### 2.1. Materials

The spelt seeds (Triticum spelta L. cv. Ostro) were purchased from a local mill in the Dolenjska region of Slovenia. 2,2-diphenyl-1-picrylhydrazyl radical (DPPH^•^) reagent, Folin–Ciocalteu reagent, Trolox, hydrochloric acid, gallocatechin, caffeic acid, p-coumaric acid, *p*-hydroxybenzoic acid, *trans*-ferulic acid, α-amylase (EC 232-565-6) from porcine pancreas (enzyme activity 5 U/mg solid), pepsin (EC 232-629-3) from porcine gastric mucosa (enzyme activity ≥ 2500 U/mg protein), and pancreatin (EC 232-468-9) from porcine pancreas (4 × USP specifications) were from Sigma-Aldrich (Steinheim, Germany). Absolute methanol, formic acid, sodium hydroxide, sodium carbonate, and calcium chloride dehydrate) were from Merck (Darmstadt, Germany). Bile bovine (EC 232-369-0) was from Millipore (ZDA). Potassium chloride, potassium dihydrogen phosphate, sodium bicarbonate, sodium chloride, and magnesium chloride hexahydrate were from Sigma-Aldrich (Steinheim, Germany). Ammonium carbonate was from Honeywell/Riedel-de Haen (Seelze, Germany). All chemicals used were analytical or HPLC grade. Working solutions were prepared using Milli-Q purified water (Milli-Q; Millipore).

For bread making: white wheat flour type 500 was obtained from Žito d.o.o. (wheat flour quality parameters: dry matter: 854.9 g/kg; water: 14.52%; ash: 0.535% on a dry basis.; wet gluten: 31.4%; dry gluten: 10.6%; gluten index (GI): 89; protein content: 13.6%; enzyme activity (FN): 366); dry yeast Di-go was purchased from Lessaffre (Logatec, Slovenia) (ingredients: live yeast cells *Saccharomyces cerevisiae*, emulsifier E491).

### 2.2. Bioprocessing of Spelt Flour

Germination of hulled spelt seeds was carried out according to Mencin et al. [9], under specific abiotic stress conditions (darkness, at 25 °C for 144 h, with the addition of 25 mM NaCl after 48 h and 50 mM sorbitol after 96 h of germination). After germination, the spelt seeds were first freeze-dried (−50 °C and 30 mTorr) and then milled with a laboratory mill (A11 basic, IKA^®^ Works, Staufen, Germany). The freeze-dried, germinated milled seeds were then fermented for 72 h at 30 °C under static conditions, the sample-to-saline ratio being 1:1.5 (10 g: 15 mL), and with the addition of 0.75 mL of *S. cerevisiae* inoculum (referred to as “germinated + fermented” flour) [2]. Enzymatic treatment of the freeze-dried and milled germinated seeds was performed at 40 °C for 4 h, with the addition of cellulase (25 U/g DW), xylanase (5 U/g DW), feruloyl esterase (10 U/g DW), protease (50 U/g DW), and α-amylase (50 U/g DW) (referred to as “germinated + enzymatic treated” flour) [12]. The bioprocessed spelt seeds were freeze-dried and stored at −20 °C.

### 2.3. Bread Preparation

A white wheat bread formulation was used for the bread making, which served as the control bread, and was prepared with 1000 g of wheat flour, 700 mL of water, 7.5 g of instant bakery yeast, 19 g of salt, and 25 mL of sunflower oil. All the ingredients were mixed in a spiral mixer Diosna SP12 (Diosna Dierks and Söhne, Osnabrűck, Germany) for 3 min in a 15 rpm bowl and at a 105 rpm spiral velocity, and then for 9 min in a 30 rpm bowl and at 210 rpm spiral velocity. After kneading, the dough rested in the mixing bowl for 30 min. Then it was divided (320 g) and put into molds for fermentation and baking. Fermentation took place in the Gostol-Gopan fermentation chamber FK (Gostol-Gopan, Nova Gorica, Slovenia) for 45 min at 30 °C and 85% relative humidity. Baking the breads began with a dosage of 0.5 L of 180 °C hot steam in a Miwe aero oven (Miwe Michael Wenz GmbH, Arnstein, Germany). The baking regime was as follows: 5 min at 230 °C, 20 min at 190 °C, and 4 min at 200 °C. Depending on the type of bioprocessed bread to be made, the commercial wheat flour was replaced with the relevant proportion of bioprocessed spelt flour, namely 5% (GFB5) or 2.5% (GFB2.5) of “germinated + fermented” spelt flour, or 1% unpasteurized (GEB1) or 5% pasteurized (GEB5P) “germinated + enzymatic treated” spelt flour. Since the addition of “germinated + enzymatic treated” spelt resulted in a poor bread quality, pasteurization of the spelt (30 min, 85 °C) was applied before the flour was used as ingredient. A correction of the dry matter content was previously taken into account. All the other ingredients of the recipe, and the protocol for bread making, were the same as for the control bread. According to our previous study, these breads had the best qualitative and sensory characteristics among the different bioprocessed breads, so we selected them to determine the bioaccessibility of their phenolics after in vitro digestion. The breads were homogenized and freeze-dried before in vitro digestion.

### 2.4. In Vitro Digestion

To evaluate the effect of bioprocessing spelt flour on the bioaccessibility of phenolics in the breads, we performed in vitro GI digestion. The simulated in vitro digestion of the breads was adapted from the method of Brodkorb et al. [15], with slight modifications. The digestion process is described in detail in our previous research [13]. Briefly, 1.0 g of each freeze-dried homogenized bread was placed in a 50 mL tube and mixed with 2.67 mL of simulated salivary fluid; α-amylase was then added to achieve 75 U/mL in the final mixture, followed by 12.5 µL of 0.3 M calcium chloride dehydrate and 666 µL of water. The oral phase lasted 2 min at 37 °C. The next phase was digestion in the stomach, whereby 4 mL of simulated gastric fluid, pepsin (2000 U/mL in the final mixture), and 2.5 µL of 0.3 M calcium chloride dehydrate were added in a strongly acidic environment (pH 3). Water was added until the volume of the mixture reached 10 mL, then the mixture was incubated at 37 °C for 2 h. The third phase comprised the addition of 8 mL of simulated intestinal fluid, pancreatin, to achieve 100 U/mL of trypsin activity in the final mixture, bile bovine to achieve 10 mM in the final mixture, and 20 µL of 0.3 M calcium chloride dehydrate to mimic intestinal digestion conditions. The mixture (pH 7, 20 mL) was incubated for 2 h at 37 °C with shaking. After digestion, the aliquots of supernatants (5 mL) were stored at 4 °C until analysis. The remaining residue was freeze-dried and stored at −20 °C until analysis.

A blank sample (no added sample) was processed and examined to discard the effect of digestive enzymes and buffers on the phenolics content and antioxidant activity. Undigested samples were the ones digested with the simulated digestive fluids without enzymes or bile. Two replicates were used for each sample used for in vitro digestion.

The bioaccessibility of the total and individual phenolics was calculated as the value of extractable phenolics of digested samples divided by the total phenolic content (extractable + bound) of undigested samples exposed to simulated digestive fluids without enzymes or bile.

(1) $$Bioaccessibility\;\left(\%\right)=\frac{\mathrm{extractable}\;\mathrm{phenolics}\;\mathrm{of}\;\mathrm{digested}\;\mathrm{samples}}{\mathrm{extractable}+\mathrm{bound}\;\mathrm{phenolics}\;\mathrm{of}\;\mathrm{undigested}\;\mathrm{samples}}\times 100$$

### 2.5. Extraction and Isolation of Phenolics

The extraction of the extractable and bound phenolics was performed according to Mencin et al. [9]. An aliquot of 1 g of homogenized and freeze-dried undigested and digested samples was extracted with absolute methanol in a ratio of 1:9 (*w*:*v*). The samples were shaken for 2 h at room temperature in the dark, and then centrifuged (9793.9× *g*, 10 min) and filtered using pore size syringe filters. These filtered supernatants contained the extractable phenolics. After the methanol extraction, the remaining solid residues were hydrolyzed with sodium hydroxide (20 mL) and shaken for 4 h at room temperature. The hydrolyzed samples were acidified to pH 3 with concentrated formic acid, then centrifuged and filtered. The filtered hydrolysates contained the bound phenolics.

To remove interfering matrix compounds, and to concentrate the phenolics, the supernatants and hydrolysates were further used for solid-phase extraction (SPE). SPE was performed according to Mencin et al. [9]. The 70% methanolic eluates from the SPE-purified extracts then represented the corresponding extractable and bound fractions of phenolics. The purified methanolic fractions thus obtained were used directly for the analysis of the total and individual phenolics and their antioxidant activity.

### 2.6. Total Phenolics Content

Total phenolics contents (TPCs) were determined using the Folin–Ciocalteu spectrophotometric method, as described by Mencin et al. [9]. Aliquots of bread samples were mixed with water and Folin–Ciocalteu reagent diluted with water 1:2 (*v*:*v*). After shaking and incubation for 5 min, a 20% sodium carbonate solution was added, and incubated for 60 min at room temperature in the dark. The absorbance was measured at 765 nm using a UV–visible spectrophotometer (model 8453; Hewlett Packard, Waldbronn, Germany). A standard curve was prepared with Trolox solution (6.75–27 µg/mL) and the final results were expressed as mg Trolox equivalents per g dry weight (mg TE/g DW). Trolox is rarely used as a standard in the Folin–Ciocalteu assay, but allows for direct comparison of data from the Folin–Ciocalteu and DPPH assays.

### 2.7. Phenolic Composition

The individual phenolic content (extractable and bound) was quantified using an HPLC system (Thermo Dionex system; Thermo Scientific, San Jose, CA, USA) equipped with a UV detector set at 280 nm and 310 nm. Separation was achieved with a C18 column (Gemini C18; 150 mm × 4.6 mm; 3 µm; Phenomenex, Torrance, CA, USA). All of the phenolics were identified using mass spectrometry (LTQ XL linear ion trap mass spectrometer; Thermo Fisher Scientific, San Jose, CA, USA) equipped with an electrospray ionizer source. The mass spectrometer was operated in negative ionization mode, using the same conditions as previously reported by Mencin et al. [9]. The phenolics were identified and quantified based on comparisons of their UV-Vis spectra and MS spectra and retention times with external standards, followed by fragmentation, as described in detail in the study by Mencin et al. [9]. The concentrations of *p*-coumaric, *trans*-ferulic, caffeic, and *p*-hydroxybenzoic acids were calculated through a calibration curve with the corresponding external standards. The concentrations were expressed as µg per g bread DW (µg/g DW). One peak was tentatively identified as the *cis*-isomer of ferulic acid, because the HPLC-MS data showed mass spectra nearly identical to those of *trans*-ferulic acid, so it was quantified using the calibration curve of *trans*-ferulic acid. For compounds that were identified without standards, quantification was carried out using similar compounds as the standards.

### 2.8. Antioxidant Activity

Quantifying the DPPH^•^ radical scavenging activity of bread extracts was performed in methanol [9]. Appropriately diluted bread extracts were mixed with 0.2 mM DPPH^•^ solution in absolute methanol and made up to 2 mL with methanol. The absorbance was measured at 520 nm using a UV–Vis spectrophotometer after 1 h of incubation at room temperature in the dark. The antioxidant activity against the DPPH^•^ radical was obtained from a calibration curve made by preparing different concentrations of a Trolox solution (2.5–10.01 µg/mL) in methanol. The final data were expressed as mg TE/g DW.

### 2.9. Statistical Analysis

All of the analyses were performed in two parallel runs, on two separate extractions. The results were subjected to one-way ANOVA, followed by the Duncan’s post-hoc test, with a significant difference of *p* < 0.05. The tests were performed using the SPSS programme, version 22 for Windows (IBM, New York, NY, USA). Principal component analysis (PCA) was performed using the OriginPro 2022b programme.

## 3. Results and Discussion

### 3.1. Total Phenolic Content

The Folin–Ciocalteu reagent can be non-specifically reduced by various matrix components, such as reducing sugars, organic acids, fatty acids, and proteins as well as phenolics [16], so we used a blank sample (no added sample) to correct for interference from the digestive enzymes and buffers.

The extractable and bound TPC contents of the undigested and digested bread samples are shown in Table 1. The undigested control bread had 0.83 mg TE/g DW of extractable TPC and 3.79 mg TE/g DW of bound TPC. The addition of bioprocessed spelt flour significantly increased the extractable and bound TPC in the undigested breads, by up to 151% (GFB5 bread), while the GEB1 bread showed no significant difference. Two studies using enzymatic bioprocessing reported large increases (up to 10-fold) in the soluble phenolics content [14,17]. However, in both studies, the authors treated wheat or rye bran by a combination of yeast fermentation and enzymatic treatment prior to its addition to the bread formulation. The bound TPC of undigested bioprocessed breads increased compared to the undigested control bread, with the highest increase observed in GEB5P bread (43%). No significant difference was observed in GFB2.5 bread.

As can be seen in Table 1, in vitro digestion had a different effect on the extractable (bioaccessible) and bound (non-bioaccessible) TPCs, compared to the digested control bread. The highest extractable TPC was determined for digested GFB5 bread, which increased by 33%. While the addition of “germinated + fermented” spelt flour to the bread formulation increased the extractable TPC, the substitution of white wheat flour with “germinated + enzymatic treated” spelt flour had no (5% substitution) or even a negative (1% substitution) effect on the extractable TPC. In contrast, the addition of “germinated + enzymatic treated” spelt flour increased the bound TPC compared to the digested control bread, while the addition of “germinated + fermented” spelt flour had no effect.

Our previous study showed that most phenolics in *Triticum* seeds are in the bound form, and able to survive upper GI digestion [13]. In the current study, the TPCs of the undigested breads showed a predominance of bound phenolics, from 67% in GFB5 bread to 83% in GEB1 bread, while in the digested breads the bound TPC ranged from 47% in GFB5 bread to 61% in GEB1 bread, over total (extractable + bound) TPC. In vitro digestion of the breads enhanced the extractable TPC, the highest increase being observed in the digested control bread (252%) and the lowest in the GFB5 bread (86%). The increase in the amount of extractable TPC in breads after digestion could be due to the fact that digestive enzymes break the chemical bonds between phenolics and structural components, thus promoting the release of bound phenolics [18]. In addition, the transition from an acidic to an alkaline environment leads to the deprotonation of hydroxyl groups of aromatic rings, which may have contributed to the increased extractable TPC in the digested breads [19]. Another possible reason could be the structure of the bioprocessed flour; germinated fermented or enzymatic treated flour is generally more digestible because of the enzymatically modified structure of starch granules, thin cell walls, and available mono- and disaccharides. Polysaccharides in the cell walls are hydrolysed by the enzymes during bioprocessing techniques, resulting in changes in the composition of soluble and insoluble dietary fibre [20]. On the other hand, the bound TPC decreased significantly in all of the digested bread samples compared to the undigested ones, with the greatest decrease (23%) observed in GFB2.5 bread and the lowest (14%) in the control bread. The decrease in the bound TPC in the digested breads indicates their partial conversion to extractable forms during GI digestion. The increase in the extractable TPC after digestion was actually higher in the control bread than in the GFB5 bread, but the absolute extractable TPC was significantly higher in the GFB5 bread (by 33%) (Table 1).

During digestion, phenolics are susceptible to various biochemical and physicochemical factors, such as the presence of enzymes, bile salts and electrolytes, and pH and ionic strength changes [21]. For example, the protonation/deprotonation of phenolics under different pH conditions affects their oxidative status and stability [22]. Despite the considerable increase in the bioaccessible TPC content with the addition of bioprocessed spelt flour, the majority of the phenolics generally remained in the non-bioaccessible form entering the colon. In the colon, fermentation of cell wall structures by bacterial enzymes is likely to facilitate the release of phenolics that were not accessible in the small intestine. Our study agrees with Anson et al. [14], who reported that the bioprocessing of wheat bran by fermentation, or by the combination of enzymatic treatment and fermentation, promotes the release of phenolics and increases their free form in wheat breads.

### 3.2. Phenolic Profile in Undigested and Digested Breads

As shown in Table 2 and Table 3, a total of nine phenolics were identified and quantified, with *trans*-ferulic acid representing the main component.

**Figure 1:**
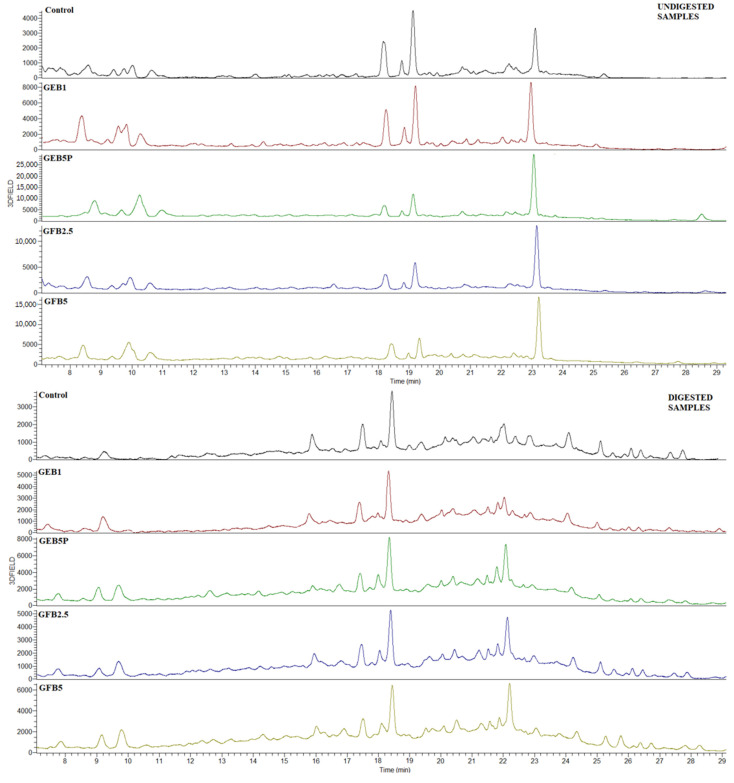
HPLC chromatograms of extractable phenolics before and after in vitro digestion of control and bioprocessed breads. Detection was achieved at 310 nm. Control: wheat bread with no added bioprocessed flour; GEB1: wheat bread enriched with 1% “germinated + enzymatic treated” spelt flour; GEB5P: wheat bread enriched with 5% pasteurized “germinated + enzymatic treated” spelt flour; GFB2.5: wheat bread enriched with 2.5% “germinated + fermented” spelt flour; GFB5: wheat bread enriched with 5% “germinated + fermented” spelt flour.

**Figure 2:**
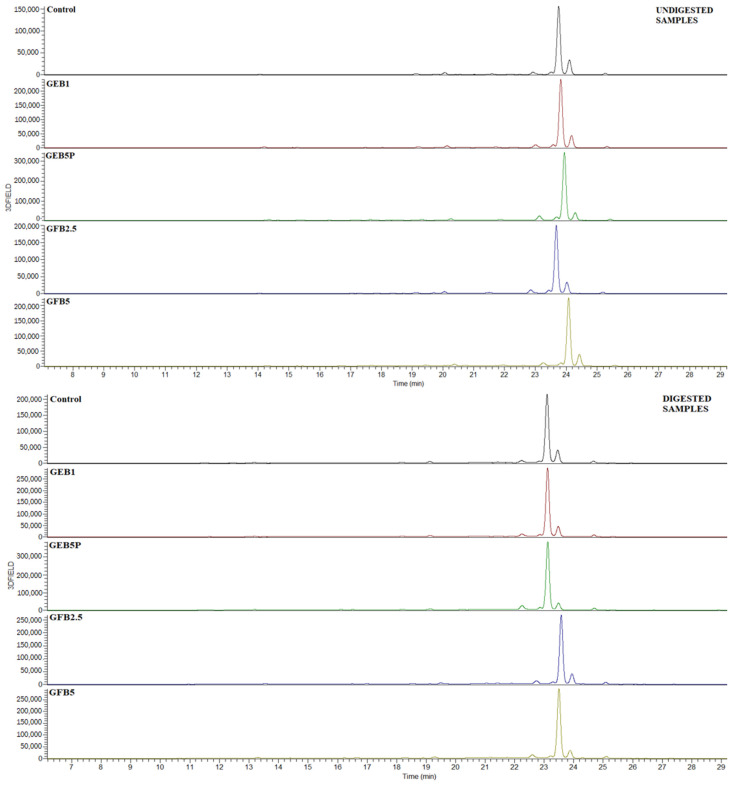
HPLC chromatograms of bound phenolics before and after in vitro digestion of control and bioprocessed breads. Detection was achieved at 310 nm. Control: wheat bread with no added bioprocessed flour; GEB1: wheat bread enriched with 1% “germinated + enzymatic treated” spelt flour; GEB5P: wheat bread enriched with 5% pasteurized “germinated + enzymatic treated” spelt flour; GFB2.5: wheat bread enriched with 2.5% “germinated + fermented” spelt flour; GFB5: wheat bread enriched with 5% “germinated + fermented” spelt flour.

Among the undigested breads, the control bread generally contained the lowest content of extractable and bound phenolic acids (Figure 1 and Figure 2). The highest increases in extractable and bound *p*-coumaric acid (36% and 448%, respectively), *trans*-ferulic acid (356% and 155%, respectively), *cis*-ferulic acid (216% and 52%, respectively), and bound caffeic acid (330%) were observed in the GEB5P bread, while the highest increases in extractable caffeic acid (238%) and extractable and bound *p*-hydroxybenzoic acid (41% and 49%, respectively) were found in the GFB5 bread compared to the undigested control bread. To summarize, the percentage of added bioprocessed spelt flour, and the type of seed pre-treatment, have a major impact on the extractable and bound phenolic acid contents in enriched breads.

Among the digested samples, almost all phenolic acids had their lowest content in the control bread. The highest content of extractable and bound phenolic acids in digested breads was found in breads enriched with 5% “germinated + fermented” (GFB5) or 5% pasteurized “germinated + enzymatic treated” spelt flour (GEB5P). The content of extractable *p*-coumaric, *trans*-ferulic, *cis*-ferulic, caffeic, and *p*-hydroxybenzoic acids increased by 7.5-fold, 3.8-fold, 3.2-fold, 3.9-fold, and 1.6-fold, respectively, in the digested GFB5 bread compared to the digested control bread. The content of bound *p*-coumaric, *trans*-ferulic, *cis*-ferulic and *p*-hydroxybenzoic acids increased by 3.9-fold, 1.8-fold, 1.3-fold, and 1.8-fold, respectively, in the GEB5P bread compared to the digested control bread, and the bound caffeic acid content increased by 2.6-fold in the GFB5 bread.

In general, extractable *p*-coumaric and *trans*-ferulic acids decreased in the breads after digestion (Table 2); the highest decrease in extractable *p*-coumaric acid (86%) was found in the control bread, and the highest decrease in extractable *trans*-ferulic acid (74%) was observed in the GEB5P bread. In contrast, the extractable *cis*-ferulic and *p*-hydroxybenzoic acids contents increased in all breads. The highest increase in extractable *cis*-ferulic and *p*-hydroxybenzoic acids content was in the GEB1 bread, by 87% and 214%, respectively, compared to the corresponding undigested samples. The extractable caffeic acid behaved differently during digestion in each bread sample, e.g., it increased by 100% in the GEB1 bread and decreased by 20% in the GFB5 bread. It should be emphasized that, although the extractable *trans*-ferulic acid content decreased after digestion, the bioprocessed breads contained a significantly higher extractable *trans*-ferulic acid content, up to 283% in the GFB5 bread, compared to the digested control bread. The decrease in the concentration of extractable phenolic acids may be caused by their enzymatic and thermal degradation, as well as by physical and chemical binding [23]. A possible reason for the loss of released phenolic acids is their heat resistance. Ferulic acid, and other hydroxycinnamic acids, decarboxylate on heat treatment to form ring-substituted styrenes/vinylbenzenes [24]. Liazid et al. [25] found that the more hydroxyl substituents, and the fewer methoxy substituents, present, the easier it is to degrade phenolic acid. Moreover, extractable phenolic acids can also be redistributed and repeatedly bound via covalent or non-covalent bonds, by dough and bread components [26,27,28]. Phenolic acids can be covalently bound to proteins and to starch molecules by the esterification of three hydroxyl groups of a glucose unit, furthermore they can be non-covalently entrapped in the hydrophobic core of polysaccharides (mainly amylose) [28].

The content of bound phenolic acids in the control and bioprocessed breads demonstrated a significant increase after GI digestion compared to the corresponding undigested sample, with the exception of bound *p*-hydroxybenzoic acid in the control bread. It is worth noting that the predominant bound *trans*-ferulic acid showed the opposite digestion trend to the TPC.

Amaya Villalva et al. [29] reported that after intestinal digestion, the free ferulic acid content was higher than the initial values for native bran bread, except for white bread enriched with 30% fermented and enzymatic treated bran, which showed a decrease compared with initial values (−47%). Skrajda-Brdak et al. [23] reported that after in vitro digestion, the levels of free phenolic acids of prepared breads were 3 to 9-fold higher than in the initial flours. Furthermore, Tian et al. [30] reported that simulated digestion significantly increased the percentage of soluble *trans*-ferulic acid for bread (3.1% to 15.4%) compared to the undigested sample. Anson et al. [14] reported that bran fermentation increased the content of free ferulic acid in the bread by 3-fold, while a combination of fermentation and enzymatic treatment of bran increased the content of free ferulic acid in the bread by 8-fold. Moreover, they reported that these bioprocessing techniques also increased the free *p*-coumaric acid content.

The reason for the increase in the bound phenolic acids content after digestion could be that digestive enzymes facilitated their extraction. On the other hand, Ti et al. [31] reported a significant increase in bound phenolic acid during the in vitro digestion of cooked polished and brown rice, which they attributed to the fact that phenolic acids are naturally embedded in the fibre-protein network and these complexes become more accessible under digestion conditions. Digestive enzymes and acidic conditions in the gastric phase weaken the network, allowing more phenolic acids to be released from the digestion residue [30]. However, the final content of these phenolic acids in bread is the result of the combined processes of their release from cell walls, degradation by enzymes of flour and microflora, thermal degradation during baking, and repeated binding by surrounding carbohydrates and proteins [23].

In general, the highest content of extractable (bioaccessible) phenolic acids was found in breads produced from bioprocessed spelt flours. The type of bioprocessed spelt flour had a lower impact on the content of the individual phenolic acids than the percentage of bioprocessed flour added. Interestingly, the content of extractable *trans*-ferulic acid in undigested breads was up to 24.0-fold higher than the extractable *p*-coumaric acid content, whereas the content of extractable *trans*-ferulic acid in digested breads was only up to 14.4-fold higher than the extractable *p*-coumaric acid content, indicating a much higher bioaccessibility of *p*-coumaric acid than of *trans*-ferulic acid. The same trend was observed when we compared extractable *trans*-ferulic acid with the content of other extractable phenolic acids. Phenolic acids that are in the bound form after digestion are considered to be non-bioaccessible [32]. Despite the fact that ferulic and *p*-coumaric acids were mainly present in the bound form, they were still the most abundant bioaccessible phenolics of the control and bioprocessed wheat breads.

The extractable fraction of the undigested and digested breads also contained various flavonoid derivatives, including apigenin hexoside pentoside I, II, III, and an unknown C-glycosyl derivative (Table 3, Figure 1). Among the undigested breads, the addition of bioprocessed spelt flour to the bread recipe significantly increased all three apigenin derivatives, with the highest increase observed in the GFB5 and GEB5P breads. On the other hand, the highest content of the C-glycosyl derivative was found in the undigested control bread.

Among the digested breads, the control bread had the lowest content of apigenin I, II, and III, while the GEB1 bread had the lowest content of the C-glycosyl derivative. The highest content of apigenin I and II was found in the GFB5 and GEB5P breads. The highest content of apigenin III was found in the GEB5P and GFB2.5 breads, and GFB5 bread had the highest content of the C-glycosyl derivative. After digestion, the content of all three apigenin derivatives decreased compared to the corresponding undigested samples. The highest decrease in apigenin I and II content was found in the digested control bread, by 45% and 53%, respectively, while for apigenin III in the GEB1 bread, the decrease was 35%. Interestingly, the content of the C-glycosyl derivative considerably increased after digestion compared to the corresponding undigested samples, (e.g., 429% in GFB5 bread), probably due to hydrolysis of complex compounds from their glycoside to aglycone forms, and also to cleavage of the C-ring and reduction of double bonds [33].

It can be concluded that it is particularly important to increase the content of extractable phenolics in bread by adding bioprocessed spelt flour, because they are lost during digestion.

### 3.3. Antioxidant Activity of Undigested and Digested Bread Samples

Phenolics are very sensitive to various environmental conditions, which means that their properties can change significantly depending on the conditions in which they occur. For this reason, the change in antioxidant activity was studied after GI digestion of breads enriched with bioprocessed spelt flour. The results of the ability of the undigested and digested control breads and the breads enriched with bioprocessed spelt flour to reduce free radicals by the DPPH^•^ method are presented in Table 1. The results of the undigested samples showed that the addition of bioprocessed spelt flour to the bread formulation significantly increased the DPPH^•^ scavenging activity of the extractable and bound fractions, with the highest increase in the GEB5P bread, by 100% for the extractable fraction and by 60% for the bound fraction.

The results for the digested breads generally showed that the addition of bioprocessed spelt flour to the bread formulation improved the DPPH^•^ scavenging activity of the extractable fraction, with the highest increase, of 88%, in the GFB5 bread. For the bound fractions of digested breads, an increase in DPPH values was found only in the GEB1 and GEB5P breads, by 5% and 18%, respectively, compared to the digested control bread. The highest proportion of antioxidant activity was found in the bound fraction. The percentage of added bioprocessed spelt flour in the bread recipe had a significant effect only on the antioxidant activity of the extractable fraction.

Interestingly, the DPPH values for the extractable fraction of the control and bioprocessed breads after digestion were lower than those before digestion, ranging from 20% for the control bread to 30% for the GEB5P bread. Ydjedd et al. [34] reported that some phenolics can be converted into different structural forms, with different chemical properties, due to their sensitivity to neutral pH, while others [34,35,36] have attributed the decrease in antioxidant activity to the decrease in the content of phenolics after digestion. Although in the present study the extractable TPCs increased after digestion, while their antioxidant activity decreased, the digested breads with higher extractable TPCs had, in general, higher antioxidant activities. However, the antioxidant activity of phenolics may also depend on their molecular structure, because hydroxycinnamic acids can ensure a greater capacity to transfer protons, and subsequently stabilizing radicals, than hydroxybenzoic acids. Phenolics can also acquire the ability to react and bind with bread matrix components, leading to a decrease in their antioxidant activity [37]. The loss of antioxidant activity may be due to synergistic combinations or interactions of different types of chemical reactions, the diffusion of water-soluble compounds, and their formation or degradation [38]. Our results are in line with the results of Chen et al. [38], who reported that the DPPH^•^ radical scavenging activity of different sesame varieties after digestion decreased compared to corresponding undigested samples. On the other hand, Chait et al. [35] reported that the DPPH values of carob phenolics in soluble free form showed a significant increase after GI digestion with respect to undigested extracts.

In contrast, the bound DPPH values increased in the control and bioprocessed breads, from 63% in the GEB5P bread to 120% in the control bread, after digestion. The difference in antioxidant activity may be attributed to the synergistic or antagonistic effect of bioactive compounds [39], pH changes, and deprotonation of hydroxyl groups on the aromatic rings of the phenolics, or to structural changes of phenolic molecules or the release of new compounds with higher antioxidant activities [40].

Several studies have reported a relationship between phenolics content and antioxidant activity [34,35,38], but very weak negative coefficients of correlation (r) between TPC and antioxidant activity (r = −0.072 and r = −0.367, respectively) were determined for our undigested and digested breads. The unclear relationship between TPC and DPPH^•^ scavenging activity may be explained by, among other things, the non-specificity of the two methods, the profile of antioxidants, and interactions between the bioactive molecules present [41].

### 3.4. Bioaccessibility of Phenolics in Bread Samples

#### 3.4.1. Bioaccessibility of Total Phenolics

In this study, an in vitro simulated digestion model was used to mimic the in vivo physiological environment. Bioaccessibility may depend not only on the availability of phenolics in the food matrix but also on the complexity of the food matrix, or on conditions in the GI tract, etc. In this context, it is very important to consider that the digestion process acts as a biological extraction system, in which the efficiency of extraction depends on the factors mentioned above.

The bioaccessibility of the TPCs in digested breads was calculated according to Equation (1), and the results are shown in Table 4. The application of external enzymes (GEB1, GEB5P) had a negative effect on the bioaccessibility of TPCs. On the other hand, the breads with the highest bioaccessibility (GFB5, GFB2.5) also showed high levels of bioaccessible (extractable) TPC (Table 1) and individual phenolic acids (Table 2). Interestingly, the control bread, with a lower content of bioaccessible TPC than the GEB5P bread, showed a significantly higher bioaccessibility. The higher bioaccessibility of the TPC in the control bread was probably due to a lower content of extractable and bound TPC in the undigested control bread and also to an increase in the extractable TPC after digestion. As expected, the bioprocessed breads had a higher initial total (extractable + bound) TPC, due to the preservation of the outer layers of spelt seeds and the improved nutritional value of spelt flour. Consequently, this contributed to a higher absolute content of bioaccessible TPC, suggesting that the use of bioprocessed whole spelt flour is a suitable strategy for this purpose. Although the absolute content of bioaccessible TPC was higher in bioprocessed breads than in the control bread, the bioaccessibility was higher in the control bread than in the bioprocessed breads. We assumed that the simple matrix of the control (white wheat) bread contributed to this result, as this sample was composed of starchy endosperm that can be easily digested by digestive enzymes to release phenolics. In contrast, the bioprocessed whole spelt flour added to the white flour modified the composition of the food matrix, making it more complex and difficult for digestive enzymes to digest, which was reflected in the lower bioaccessibility of the TPC compared to the control bread. The bioprocessing of spelt seeds increased the bioaccessibility of TPC, which is probably due to the action of hydrolytic enzymes that can degrade the bran cell wall [7].

Our results are in accordance with Angioloni and Collar [42], who reported that the bioaccessibility of TPC for wheat bread was 58%. Similarly, Anson et al. [14] reported a higher bioaccessibility of phenolics in white bread (5%) compared to whole wheat bread (1%). Dietary fibre, resistant starch, resistant protein, and Maillard compounds resistant to the action of digestive enzymes, may reduce the breads’ phenolics bioaccessibility [42].

#### 3.4.2. Bioaccessibility of Individual Phenolics

Among individual phenolics, *trans*-ferulic acid showed the lowest bioaccessibility (Table 4). Angelino et al. [7] reported that ferulic acid appears to have a lower bioaccessibility than other phenolic acids. They suggested as a reason, the different distribution of phenolics in the extractable and bound forms; *trans*-ferulic acid showed only up to 2.8% bioaccessibility in the GFB5 bread. The bioaccessibility of *p*-hydroxybenzoic acid was up to 61.1% in the GEB1 bread. In the GFB2.5 and GFB5 breads, the bioaccessibility of *p*-coumaric, *trans*-ferulic, and *cis*-ferulic acids was found to be higher than in the other digested breads. In the GFB5 bread, the bioaccessibility of *p*-coumaric, *trans*-ferulic and *cis*-ferulic acids was 179%, 115% and 130%, respectively, higher than in the control bread. Enrichment of bread with bioprocessed spelt flour resulted in the higher bioaccessibility of caffeic acid, which was greatest in the GEB1 bread.

The highest bioaccessibility of apigenin II and III was found in the GFB2.5 bread, while apigenin I was found in the GFB 5 bread, which also showed the highest content of extractable apigenin I. Interestingly, the highest bioaccessibility of the unknown C-glycosyl derivative was found in the control bread, due to the greater proportion of the extractable form, which significantly increased after digestion (by 227%). The lowest bioaccessibility of apigenin I and III was found in the GEB1 bread, while for apigenin II it was found in the control bread, which also showed the highest decrease in the content of the extractable form after digestion. We found the lowest bioaccessibility of the C-glycosyl derivative in the GEB5P bread.

Anson et al. [14] reported a large variation in the bioaccessibility of ferulic acid in different types of bread. They also showed that a combination of fermentation and enzymatic treatment increased the bioaccessibility of ferulic acid 5-fold compared to bread with native bran. In addition, the bioaccessibility of *p*-coumaric acid in the breads was also increased (about 2-fold) by bioprocessing the bran. In another study, conducted by Hemery et al. [43], a 10.2% ferulic acid bioaccessibility was found in white bread, which was significantly higher than in whole grain bread and bran enriched breads. Koistinen et al. [17] determined a significantly higher ferulic acid bioaccessibility in wheat bread enriched with bioprocessed rye bran compared to similar bread enriched with native rye (88% and 51%, respectively). The great increase in bioaccessibility of phenolic acids in bioprocessed breads may be due to the hydrolysis of various wheat fiber polymers by the hydrolytic enzymes, which can lead to a structural breakdown of the bran cell walls [7].

It should be noted that although we improved the content of bioaccessible (extractable) phenolic acids and their bioaccessibility, the majority of phenolic acids remain in the bound form, entering the colon intact, where they are fermented by colonic microbiota. Many scientists have pointed out that the prevention of colon cancer by the consumption of cereals may be associated with their bound phenolics content. In relation to these studies, it could be suggested that, although the majority of bound phenolic acids are not released from their food matrix during GI digestion, they may still have a health-promoting effect [44].

Overall, studies on the bioaccessibility of individual phenolics in breads suggest that the use of various bioprocessing techniques is a promising strategy for increasing the amount of bioaccessible phenolics in bread. The incorporation of bioprocessed flour into bakery products is therefore a cost effective strategy for improving its nutritional properties.

### 3.5. Correlation Analysis and Principal Component Analysis

Correlations between the content of extractable phenolic acids in the breads before and after digestion were calculated. The r-values were 0.961, 0.907, 0.806 and 0.789 for extractable *trans*-ferulic, *cis*-ferulic, caffeic, and *p*-hydroxybenzoic acids, respectively. The strong positive correlation between the extractable phenolic acids content in the undigested and digested breads indicates that a higher content of these phenolic acids in the undigested breads results in a higher content of bioaccessible phenolic acids in the digested breads. It is therefore important to increase the extractable phenolics content in breads before the digestion process. On the other hand, the content of extractable *p*-coumaric acid in the undigested breads had a negligible (r = 0.093) effect on the content of bioaccessible *p*-coumaric acid in the digested breads.

**Figure 3:**
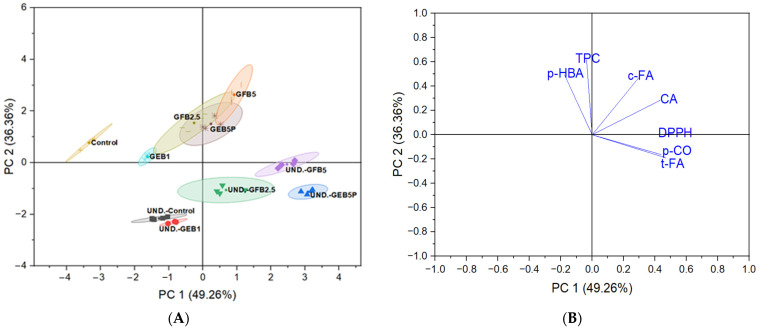
Principal component analysis score plot (**A**) and loading plot (**B**) describing the relationships among different extractable biochemical parameters of the undigested and digested breads. Control: wheat bread with no added bioprocessed flour; GEB1: wheat bread enriched with 1% “germinated + enzymatic treated” spelt flour; GEB5P: wheat bread enriched with 5% pasteurized “germinated + enzymatic treated” spelt flour; GFB2.5: wheat bread enriched with 2.5% “germinated + fermented” spelt flour; GFB5: wheat bread enriched with 5% “germinated + fermented” spelt flour. UND.: undigested. Biochemical parameters: *p*-CO: *p*-coumaric acid; t-FA: *trans*-ferulic acid; c-FA: *cis*-ferulic acid; CA: caffeic acid; *p*-HBA: *p*-hydroxybenzoic acid; TPC: total phenolic content; DPPH: DPPH^•^ scavenging activity.

Principal component analysis (PCA) was performed to use various biochemical parameters in extractable form (TPC, DPPH^•^, *p*-coumaric, *trans*-ferulic, *cis*-ferulic, caffeic, and *p*-hydroxybenzoic acids) to classify and distinguish the undigested from the digested breads (Figure 3). A total of 85.7% of the data variation was explained by the relation between principal component 1 (PC1) and principal component 2 (PC2). PC1 explained 49.3% of the data variance due to the amounts of DPPH^•^ assay, *trans*-ferulic acid, *p*-coumaric acid, and caffeic acid. PC2 explained 36.4% of the data variance based on the amounts of TPC, *cis*-ferulic acid, and *p*-hydroxybenzoic acid. DPPH^•^ scavenging activity, *p*-coumaric acid, and *trans*-ferulic acid were concentrated in the data group of the undigested GFB5 and GEB5P breads, whereas TPC, *cis*-ferulic acid, and *p*-hydroxybenzoic acid were concentrated in the data group of the digested GFB2.5, GEB5P, and GFB5 breads. PC2 separated the undigested breads from the digested breads. The digested breads were associated with higher extractable TPC, *cis*-ferulic, and *p*-hydroxybenzoic acids contents, while the undigested breads were associated with higher DPPH values, and *p*-coumaric and *trans*-ferulic acid contents.

Overall, PCA showed that the digestion process negatively affected the content of extractable (bioaccessible) *p*-coumaric and *trans*-ferulic acids, as well as antioxidant activity against DPPH^•^ radicals. On the other hand, the extractable (bioaccessible) TPC, *p*-hydroxybenzoic acid, and *cis*-ferulic acid contents increased in the bread samples after the digestion process. We can also conclude that the addition of a higher percentage of bioprocessed spelt flour to the bread formulation improved the content of extractable TPC, individual phenolic acids, and their antioxidant activity in the undigested and digested bioprocessed breads compared to the corresponding control breads.

## 4. Conclusions

To summarize, the addition of bioprocessed spelt flour to a wheat bread formulation, and in vitro GI digestion, significantly affected the extractable and bound TPC, phenolic acids, flavonoids, and antioxidant activity against DPPH^•^ radicals. The addition of “germinated + fermented” spelt flour to the bread formulation increased the extractable TPC in the digested breads in contrast to the “germinated + enzymatic treated” spelt flour. The DPPH values for the extractable fraction of the control and bioprocessed breads decreased after digestion. The highest content of extractable and bound phenolic acids in the digested breads was found in the breads enriched with 5% “germinated + fermented” or 5% pasteurized “germinated + enzymatic treated” spelt flour. In general, extractable *p*-coumaric and *trans*-ferulic acids in the breads decreased after digestion, while the extractable *cis*-ferulic and *p*-hydroxybenzoic acids content increased in breads after digestion. Although the extractable *trans*-ferulic acid content decreased after digestion, the bioprocessed breads contained a significantly higher extractable *trans*-ferulic acid content, up to 283% in the GFB5 bread compared to the digested control bread. Among the digested breads, the control bread and the GFB5 and GFB2.5 breads showed the highest bioaccessibility of TPC, while the absolute content of extractable (bioaccessible) TPC in the bioprocessed breads was much higher than in the control bread. Among individual phenolics, *trans*-ferulic acid had the lowest bioaccessibility, up to 2.8%, while the bioaccessibility of *p*-hydroxybenzoic acid was up to 61.2%. In the GFB2.5 and GFB5 breads, the bioaccessibilities of *p*-coumaric, *trans*-ferulic, and *cis*-ferulic acids were found to be higher than in the other digested breads. Nevertheless, many of the bread phenolics remained undigestible and reached the colon, where they are metabolized to exert their health benefits.

Increasing the content of bioaccessible phenolics and their bioaccessibility from a daily consumed food like bread could have an important impact on consumer health. According to the results of a technological and sensory evaluation of prepared enriched breads (currently submitted paper), and according to the results of the current research, we would strongly recommend replacing 5% white flour with flour made from germinated and fermented whole spelt grain.

## Figures and Tables

**Table 1:** Total phenolic content (TPC) and antioxidant activity against DPPH^•^ radical (DPPH) of undigested and digested control and bioprocessed breads in extractable and bound fractions.

	TPC		DPPH	
	mg TE/g DW		mg TE/g DW	
Sample	Extractable	Bound	Extractable	Bound
***Undigested***				
Control	0.83 ± 0.04 ^a^	3.79 ± 0.72 ^BC^	0.10 ± 0.01 ^b^	0.15 ± 0.02 ^A^
GEB1	1.06 ± 0.03 ^a^	5.04 ± 0.11 ^E^	0.12 ± 0.01 ^c^	0.20 ± 0.02 ^C^
GEB5P	1.66 ± 0.05 ^b^	5.43 ± 0.39 ^E^	0.20 ± 0.00 ^e^	0.24 ± 0.02 ^D^
GFB2.5	1.54 ± 0.05 ^b^	4.20 ± 0.14 ^CD^	0.14 ± 0.01 ^d^	0.18 ± 0.01 ^B^
GFB5	2.08 ± 0.04 ^c^	4.27 ± 0.09 ^D^	0.19 ± 0.01 ^e^	0.20 ± 0.01 ^C^
***Digested***				
Control	2.92 ± 0.24 ^e^	3.26 ± 0.13 ^A^	0.08 ± 0.02 ^a^	0.33 ± 0.01 ^E^
GEB1	2.64 ± 0.09 ^d^	4.10 ± 0.21 ^CD^	0.09 ± 0.00 ^ab^	0.38 ± 0.01 ^F^
GEB5P	3.12 ± 0.28 ^e^	4.24 ± 0.17 ^D^	0.14 ± 0.01 ^d^	0.39 ± 0.01 ^F^
GFB2.5	3.44 ± 0.40 ^f^	3.24 ± 0.14 ^A^	0.13 ± 0.02 ^cd^	0.33 ± 0.01 ^E^
GFB5	3.87 ± 0.23 ^g^	3.47 ± 0.12 ^AB^	0.15 ± 0.01 ^d^	0.33 ± 0.01 ^E^

Results are expressed as the mean ± standard deviation. Means with different small letters within a column indicate significant differences between the content of extractable phenolics (*p* < 0.05; Duncan’s multiple range test). Means with different capital letters within a column indicate significant differences between the content of bound phenolics (*p* < 0.05; Duncan’s multiple range test). mg TE/g DW: mg Trolox equivalents per g dry weight. Control: wheat bread with no added bioprocessed flour; GEB1: wheat bread enriched with 1% “germinated + enzymatic treated” spelt flour; GEB5P: wheat bread enriched with 5% pasteurized “germinated + enzymatic treated” spelt flour; GFB2.5: wheat bread enriched with 2.5% “germinated + fermented” spelt flour; GFB5: wheat bread enriched with 5% “germinated + fermented” spelt flour.

**Table 2:** Content of extractable and bound phenolic acids of both undigested and digested control and bioprocessed breads.

	Phenolic Acids (µg/g DW)									
	*p*-Coumaric Acid		*trans*-Ferulic Acid		*cis*-Ferulic Acid		Caffeic Acid		*p*-Hydroxybenzoic Acid	
Breads	Extractable	Bound	Extractable	Bound	Extractable	Bound	Extractable	Bound	Extractable	Bound
***Undigested***										
Control	0.28 ± 0.01 ^f^	1.30 ± 0.13 ^A^	1.72 ± 0.06 ^c^	49.44 ± 4.20 ^A^	0.31 ± 0.01 ^a^	11.27 ± 1.07 ^A^	0.13 ± 0.01 ^b^	0.23 ± 0.01 ^A^	1.12 ± 0.02 ^c^	2.35 ± 0.13 ^D^
GEB1	0.23 ± 0.01 ^d^	2.72 ± 0.09 ^C^	2.52 ± 0.11 ^e^	88.15 ± 0.60 ^D^	0.38 ± 0.01 ^a^	16.91 ± 0.62 ^D^	0.13 ± 0.01 ^b^	0.43 ± 0.02 ^BC^	0.36 ± 0.01 ^a^	1.49 ± 0.08 ^B^
GEB5P	0.38 ± 0.01 ^h^	7.13 ± 0.26 ^G^	7.85 ± 0.69 ^h^	125.89 ± 4.87 ^H^	0.98 ± 0.03 ^e^	17.14 ± 0.45 ^D^	0.33 ± 0.01 ^d^	0.99 ± 0.03 ^E^	0.51 ± 0.02 ^b^	2.97 ± 0.10 ^E^
GFB2.5	0.25 ± 0.02 ^e^	3.14 ± 0.06 ^D^	6.07 ± 0.51 ^f^	75.43 ± 1.86 ^B^	0.72 ± 0.05 ^c^	13.88 ± 0.10 ^B^	0.24 ± 0.02 ^c^	0.39 ± 0.02 ^B^	1.25 ± 0.05 ^d^	1.16 ± 0.02 ^A^
GFB5	0.30 ± 0.01 ^g^	3.59 ± 0.03 ^E^	7.02 ± 0.06 ^g^	79.35 ± 1.24 ^BC^	0.79 ± 0.03 ^d^	15.25 ± 0.37 ^C^	0.44 ± 0.03 ^e^	0.68 ± 0.02 ^D^	1.58 ± 0.03 ^f^	3.50 ± 0.12 ^F^
***Digested***										
Control	0.04 ± 0.01 ^a^	2.10 ± 0.13 ^B^	0.47 ± 0.01 ^a^	80.72 ± 3.02 ^C^	0.43 ± 0.03 ^b^	15.77 ± 0.61 ^C^	0.09 ± 0.01 ^a^	0.48 ± 0.00 ^C^	1.81 ± 0.12 ^g^	2.09 ± 0.10 ^C^
GEB1	0.09 ± 0.01 ^b^	3.71 ± 0.35 ^E^	0.88 ± 0.02 ^b^	113.38 ± 2.22 ^G^	0.71 ± 0.02 ^c^	18.33 ± 0.64 ^E^	0.26 ± 0.02 ^c^	0.65 ± 0.03 ^D^	1.13 ± 0.04 ^c^	3.01 ± 0.03 ^E^
GEB5P	0.15 ± 0.01 ^c^	8.15 ± 0.29 ^H^	2.06 ± 0.14 ^cd^	143.77 ± 2.15 ^I^	1.21 ± 0.05 ^f^	20.29 ± 0.53 ^F^	0.35 ± 0.02 ^d^	1.14 ± 0.02 ^F^	1.55 ± 0.08 ^f^	3.79 ± 0.02 ^G^
GFB2.5	0.24 ± 0.01 ^de^	3.55 ± 0.13 ^E^	1.87 ± 0.12 ^c^	96.89 ± 2.52 ^E^	0.98 ± 0.07 ^e^	16.78 ± 0.44 ^D^	0.26 ± 0.01 ^c^	0.98 ± 0.10 ^E^	1.45 ± 0.01 ^e^	3.06 ± 0.09 ^E^
GFB5	0.30 ± 0.02 ^g^	5.24 ± 0.18 ^F^	2.45 ± 0.04 ^de^	108.43 ± 1.63 ^F^	1.37 ± 0.09 ^g^	19.04 ± 0.73 ^E^	0.35 ± 0.01 ^d^	1.23 ± 0.06 ^G^	2.87 ± 0.04 ^h^	3.67 ± 0.07 ^G^

Values are means ± standard deviation. Means followed by different small letters within a column indicate significant differences between the content of extractable phenolic acids (*p* < 0.05; Duncan’s multiple range test). Means followed by different capital letters within a column indicate significant differences between the content of bound phenolic acids (*p* < 0.05; Duncan’s multiple range test). Control: wheat bread with no added bioprocessed flour; GEB1: wheat bread enriched with 1% “germinated + enzymatic treated” spelt flour; GEB5P: wheat bread enriched with 5% pasteurized “germinated + enzymatic treated” spelt flour; GFB2.5: wheat bread enriched with 2.5% “germinated + fermented” spelt flour; GFB5: wheat bread enriched with 5% “germinated + fermented” spelt flour.

**Table 3:** Content of extractable flavonoids of both undigested and digested control and bioprocessed breads.

	Flavonoids (µg/g DW)			
	Apigenin			Unknown C-glycosyl Derivative
	I	II	III	
Breads	Extractable	Extractable	Extractable	Extractable
***Undigested***				
Control	1.22 ± 0.01 ^c^	0.32 ± 0.02 ^c^	1.45 ± 0.02 ^d^	0.22 ± 0.01 ^c^
GEB1	1.43 ± 0.01 ^e^	0.48 ± 0.01 ^e^	1.66 ± 0.03 ^f^	0.19 ± 0.01 ^b^
GEB5P	1.73 ± 0.03 ^g^	0.60 ± 0.00 ^f^	1.72 ± 0.02 ^g^	0.17 ± 0.00 ^b^
GFB2.5	1.56 ± 0.10 ^f^	0.47 ± 0.02 ^e^	1.57 ± 0.10 ^e^	0.15 ± 0.01 ^a^
GFB5	1.76 ± 0.06 ^g^	0.69 ± 0.02 ^g^	1.65 ± 0.04 ^f^	0.14 ± 0.00 ^a^
***Digested***				
Control	0.67 ± 0.02 ^a^	0.15 ± 0.00 ^a^	1.05 ± 0.03 ^a^	0.72 ± 0.01 ^g^
GEB1	0.81 ± 0.02 ^b^	0.23 ± 0.01 ^b^	1.08 ± 0.02 ^a^	0.51 ± 0.01 ^d^
GEB5P	1.35 ± 0.08 ^d^	0.39 ± 0.02 ^d^	1.26 ± 0.03 ^c^	0.54 ± 0.03 ^e^
GFB2.5	1.19 ± 0.08 ^c^	0.33 ± 0.01 ^c^	1.29 ± 0.06 ^c^	0.70 ± 0.01 ^f^
GFB5	1.36 ± 0.04 ^d^	0.38 ± 0.02 ^d^	1.20 ± 0.02 ^b^	0.74 ± 0.02 ^h^

Values are means ± standard deviation. Means followed by different letters within a column indicate significant differences between the content of extractable flavonoids (*p* < 0.05; Duncan’s multiple range test). Control: wheat bread with no added bioprocessed flour; GEB1: wheat bread enriched with 1% “germinated + enzymatic treated” spelt flour; GEB5P: wheat bread enriched with 5% pasteurized “germinated + enzymatic treated” spelt flour; GFB2.5: wheat bread enriched with 2.5% “germinated + fermented” spelt flour; GFB5: wheat bread enriched with 5% “germinated + fermented” spelt flour.

**Table 4:** Percentage of bioaccessibility of total phenolic content (TPC) and individual phenolics from control and bioprocessed breads after in vitro digestion.

	Bioaccessibility (%)									
Breads	TPC	*p*-Coumaric Acid	*trans*-Ferulic Acid	*cis*-Ferulic Acid	Caffeic Acid	*p*-Hydroxybenzoic Acid	Apigenin			Unknown C-glycosyl Derivative
							I	II	III	
Control	61.5 ^b^	2.8 ^b^	1.3 ^b^	3.7 ^a^	26.3 ^a^	52.0 ^b^	36.2 ^b^	28.2 ^a^	44.5 ^b^	64.0 ^e^
GEB1	43.3 ^a^	3.0 ^b^	1.0 ^a^	4.1 ^b^	46.6 ^d^	61.2 ^d^	32.4 ^a^	29.2 ^b^	34.6 ^a^	33.7 ^b^
GEB5P	44.1 ^a^	2.0 ^a^	1.5 ^c^	6.7 ^c^	26.1 ^a^	44.5 ^a^	43.7 ^c^	31.7 ^c^	35.9 ^a^	22.4 ^a^
GFB2.5	59.9 ^b^	7.1 ^c^	2.3 ^d^	6.7 ^c^	42.1 ^c^	60.2 ^d^	50.2 ^d^	42.0 ^d^	46.6 ^c^	45.3 ^c^
GFB5	60.9 ^b^	7.8 ^c^	2.8 ^e^	8.5 ^d^	31.4 ^b^	56.4 ^c^	53.4 ^e^	31.6 ^c^	43.7 ^b^	61.8 ^d^

Means followed by the different letters within a column indicate significant difference between the bioaccessibility (*p* < 0.05; Duncan’s multiple range test). Control: wheat bread with no added bioprocessed flour; GEB1: wheat bread enriched with 1% “germinated + enzymatic treated” spelt flour; GEB5P: wheat bread enriched with 5% pasteurized “germinated + enzymatic treated” spelt flour; GFB2.5: wheat bread enriched with 2.5% “germinated + fermented” spelt flour; GFB5: wheat bread enriched with 5% “germinated + fermented” spelt flour.

## Data Availability

All data are contained in this article.
